# Interaction between *MyD88*, *TIRAP* and *IL1RL1* against *Helicobacter pylori* infection

**DOI:** 10.1038/s41598-020-72974-9

**Published:** 2020-09-28

**Authors:** Andrea Fulgione, Marina Papaianni, Paola Cuomo, Debora Paris, Marco Romano, Concetta Tuccillo, Letizia Palomba, Chiara Medaglia, Massimiliano De Seta, Nicolino Esposito, Andrea Motta, Antonio Iannelli, Domenico Iannelli, Rosanna Capparelli

**Affiliations:** 1 Department of Agriculture Sciences, University of Naples “Federico II”, Via Università, 100, 80055 Portici, Naples, Italy; 2 Istituto Zooprofilattico Sperimentale del Mezzogiorno, Via Salute, 2, 80055 Portici, Naples, Italy; 3 Institute of Biomolecular Chemistry, National Research Council, Via Campi Flegrei, 34, 80078 Pozzuoli, Naples, Italy; 4 Hepatogastroenterology Unit, Department of Precision Medicine, University of Campania “Luigi Vanvitelli”, via Pansini, 5, 80131 Naples, Italy; 5 Department of Biomolecular Sciences, University of Urbino “Carlo Bo”, Via Santa Chiara, 27, 61029 Urbino, Italy; 6 Department of Microbiology and Molecular Medicine, University of Geneva Medical School, Rue du Général-Dufour, 24, 1211 Genève 4, Switzerland; 7 Fondazione Evangelica Betania, Via Argine, 604, 80147 Naples, Italy; 8 Université Côte D’Azur, Campus Valrose, Batiment L, Avenue de Valrose, 28, 06108 Nice CEDEX 2, France; 9 Centre Hospitalier Universitaire de Nice - Digestive Surgery and Liver Transplantation Unit, Archet 2 Hospital, Route Saint-Antoine de Ginestière 151, CS 23079, 06202 Nice CEDEX 3, France; 10 Inserm, U1065, Team 8 “Hepatic Complications of Obesity and Alcohol”, Route Saint Antoine de Ginestière 151, BP 2 3194, 06204 Nice CEDEX 3, France

**Keywords:** Genetic predisposition to disease, Metabolomics

## Abstract

The Toll-interleukin 1 receptor superfamily includes the genes interleukin 1 receptor-like 1 (*IL1RL1*), Toll like receptors (*TLRs*), myeloid differentiation primary-response 88 (*MyD88*), and MyD88 adaptor-like (*TIRAP*). This study describes the interaction between *MyD88, TIRAP* and *IL1RL1* against *Helicobacter pylori* infection. Cases and controls were genotyped at the polymorphic sites *MyD88* rs6853, *TIRAP* rs8177374 and *IL1RL1* rs11123923. The results show that specific combinations of *IL1RL1*-*TIRAP* (AA-CT; P: 2,8 × 10^–17^) and *MyD88*-*TIRAP*-*IL1RL1* (AA-CT-AA; P: 1,4 × 10^–8^) – but not MyD88 alone—act synergistically against *Helicobacter pylori.* Nuclear magnetic resonance (NMR) clearly discriminates cases from controls by highlighting significantly different expression levels of several metabolites (tyrosine, tryptophan, phenylalanine, branched-chain amino acids, short chain fatty acids, glucose, sucrose, urea, etc.). NMR also identifies the following dysregulated metabolic pathways associated to *Helicobacter pylori* infection: phenylalanine and tyrosine metabolism, pterine biosynthesis, starch and sucrose metabolism, and galactose metabolism. Furthermore, NMR discriminates between the cases heterozygous at the *IL1RL1* locus from those homozygous at the same locus. Heterozygous patients are characterized by high levels of lactate, and *IL1RL1*—both associated with anti-inflammatory activity—and low levels of the pro-inflammatory molecules IL-1β, TNF-α, COX-2, and IL-6.

## Introduction

*Helicobacter pylori* (*H. pylori*) is a Gram-negative, microaerophilic bacterium that colonizes the human stomach, and in most instances causes chronic gastritis. Though about half of the world population is infected with *H. pylori*, only < 1% of infected patients develop peptic ulcer, gastric cancer, or lymphoma^1^. The virulence of the bacterium is in fact dependent upon several factors, especially its potential to produce toxins^2^, and the different routes of infection: vertical transmission (from parents to child) curbs pathogen virulence, while horizontal transmission (from one individual to another unrelated) breaks up the reduced virulence accumulated by the pathogen in the course of the co-evolution with the previous host^1^.

Notably, there is evidence that *H. pylori* might be associated with extra gastric diseases—Alzheimer’s disease^3^, coronary heart disease^4^, atherosclerosis^5^—and, at the same time, might protect against other diseases: asthma and allergy^6^, esophageal adenocarcinoma, Barrett’s esophagus, and gastroesophageal reflux^7^. Further, pathogen eradication with antibiotics can alter the gut microbiome and foster obesity or type 2 diabetes^8^. These findings indicate the importance of knowing risks and advantages associated with *H. pylori* eradication.

The members of the Toll-interleukin 1 receptor (TIR) superfamily are all characterized by the presence of the TIR domain. The superfamily includes interleukin 1 receptor-like 1 (*IL1RL1*) (also known as ST2), the Toll like receptors (*TLRs*), the adaptor molecule myeloid differentiation primary-response protein 88 (*MyD88*) and the MyD88 adaptor-like *TIRAP* (also known as *MAL*). *TLRs* recognize pathogen associated molecular patterns (PAMPs), with *H. pylori* being recognized by several TLRs^9^. Following ligand binding, TLRs dimerize, go through a conformation change and—via their TIR domain – engage the adaptor proteins MyD88 and TIRAP, which trigger a signal cascade leading to NF-kB activation and production of cytokines^10^. While the majority of the TIR family members activate NF-kB, *IL1RL1* inhibits NF-kB activation, as demonstrated by *IL1RL1*-deficient mouse macrophages, which produce higher levels of pro-inflammatory cytokines when challenged with lipopolysaccharides (LPS)^11^. *IL1RL1* exerts its inhibitory activity sequestering the adaptor molecules MyD88 and TIRAP through the TIR domain^11^.

It is rare for genes to act alone. In most cases they form networks, highly flexible and adaptable^12^. The present study shows that *MyD88, TIRAP* and *IL1RL1*—all members of the same pathway^13^ and the first two physically associated^14^—confer resistance against *H. pylori* infection acting in concert. While *MyD88* alone is unable to confer resistance, specific combinations of *MyD88* and *TIRAP* and of *MyD88, TIRAP* and *IL1RL1* act synergistically against *H. pylori*. The phenomenon of gene interaction is generally referred to as epistasis. Since this term has more than one meaning^15^, here we prefer using the unambiguous expression “gene interaction”.

Nuclear magnetic resonance (NMR)–based metabolomics is commonly used to identify metabolic pathways and to discriminate between specific metabolic phenotypes^16,17^. Here, NMR uncovers a potential crosstalk between metabolites and genes, and specific host pathways dysregulated by *H. pylori*.

## Results

### Interactions between *MyD88, TIRAP*, and *IL1RL1*

*MyD88* and *TIRAP* interact against *H. pylori* infection^18^. We searched for potential proteins interacting with the MyD88 and TIRAP proteins using the STRING database (https://string-db.org). The confidence level and maximum number of interacting proteins were set at the 0.4 and 5, respectively.

**Figure 1:**
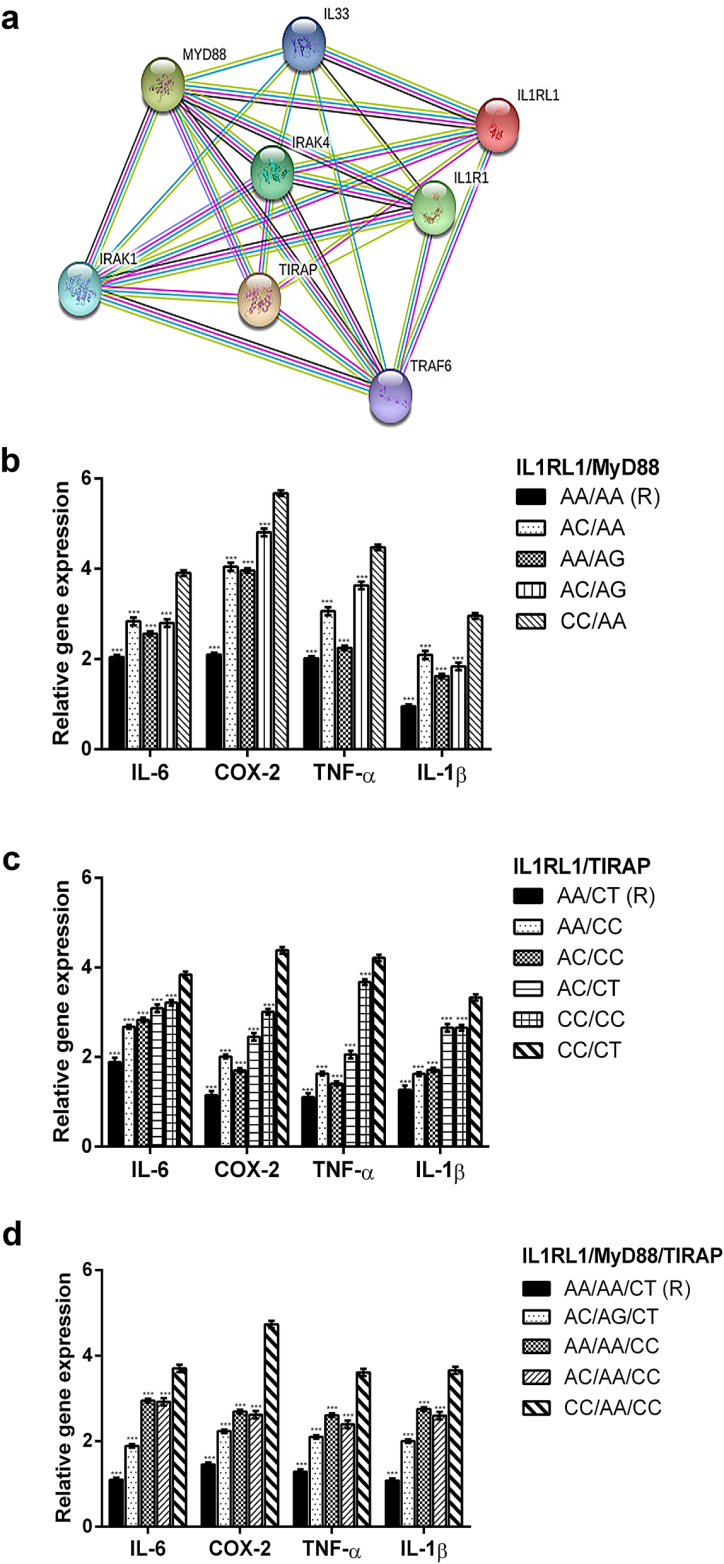
(**a**) MyD88, TIRAP and IL1RL1 interaction according to the STRING program; (**b–d**) Expression levels of IL-6, COX-2, TNF-α and IL-1β in patients with different combinations of IL1RL1, MyD88 and TIRAP. Each value represents the mean ± SD of 6 samples tested in triplicate.

STRING database provided evidence that *IL1RL1* interacts with both *MyD88* and *TIRAP* (Fig. 1a). This conclusion is validated by current literature^11,19^. The samples (cases and controls) from an earlier study^18^ were therefore used to test whether *IL1RL1* is associated with *H. pylori* infection, along with *MyD88* and *TIRAP*.

The *IL1RL1* SNP rs 11123923 was chosen to study since unique to have a rare allele frequency > 1%^20^ out of the 159 *IL1RL1* SNPs identified by sequencing 45 cases and as many control samples (Supplementary Table S1).

The genes *MyD88, TIRAP*, and *IL1RL1* were first tested individually for association with *H. pylori* infection. *TIRAP* (OR: 0.50; *P*: 3.7 × 10^–6^) and *IL1RL1* (OR: 0.59; *P*: 1.2 × 10^–4^)—but not *MyD88* (OR: 0.98; *P*: 0.95)—were associated with resistance to *H. pylori* infection (Table 1).

**Table 1:** Association between *IL1RL1* rs11123923, *MyD88* rs6853 and *TIRAP* rs8177374 polymorphic sites and *H. pylori* infection.

Genes	Status	Number of individuals in each genotype			Total	HWE (P)	Allelic frequency		OR (CI)^a^	*P* value
							Co	Ra		
		AA	AC	CC						
*IL1RL1*	Cases	138	258	102	498	0.86 (0.35)	0.54	0.46	AA vs AC 0.59 (0.46–0.77)	1.2 × 10^–4^
	Controls	297	333	72	702	2.31 (0.12)	0.66	0.34		
		AA	AG	GG						
*MyD88*	Cases	312	186	0	498	26.26 (3 × 10^–7^)	0.81	0.19	AG vs AA 0.98 (0.77–1.25)	0.95
	Controls	421	254	27	702	2.24 (0.13)	0.78	0.22		
		CC	CT	TT						
*TIRAP*	Cases	421	77	0	498	3.5 (0.061)	0.92	0.08	CT vs CC 0.50 (0.37–0.67)	3.7 × 10^–6^
	Controls	508	184	10	702	2.15 (0.14)	0.85	0.15		

*Co* common allele (*IL1RL1*: A; *MyD88*: A; *TIRAP*: C), *Ra* rare allele (*IL1RL1*: C; *MyD88*: G; *TIRAP*: T). ^a^ CI (confidence intervals) and *P* values were calculated with the Fisher’s exact test.

The study displayed additional interactions: between the genotypes *TIRAP*(CT)/*MyD88*(AG) vs *TIRAP*(CC)/*MyD88*(AA) (OR: 0.20; *P*: 9.8 × 10^–9^); *IL1RL1*(AA)/*MyD88*(AA) vs *IL1RL1*(CC)/*MyD88*(AA) (OR: 0.25; *P*: 5.9 × 10^–8^); *IL1RL1*(AA)/*TIRAP*(CT) vs *IL1LR1*(CC)/*TIRAP*(CC) (OR: 0.10; *P*: 2.8 × 10^–17^); *IL1RL1*(AA)/*MyD88*(AA)/*TIRAP*(CT) vs *IL1LR1*(CC)/*MyD88*(AA)/*TIRAP*(CC) (OR: 0.14; *P*: 1.4 × 10^–8^) (Table 2).

**Table 2:** Interaction between the *IL1RL1* rs11123923, *MyD88* rs6853 and *TIRAP* rs8177374 polymorphic sites and *H. pylori* infection.

Interactions	OR^a^	*P* value
**Allelic interactions**		
*IL1RL1* (AA vs AC)	0.59	1.2 × 10^–4^
*MyD88* (AG vs AA)	0.98	0.95
*TIRAP* (CT vs CC)	0.50	3.7 × 10^–6^
**Intergenic interactions**		
*IL1RL1*(AA)/*MyD88*(AA) vs *IL1RL1*(CC)/*MyD88*(AA)	0.25	5.9 × 10^–8^
*IL1RL1*(AA)/*MyD88*(AG) vs *IL1RL1*(CC)/*MyD88*(AA)	0.32	2.5 × 10^–5^
*TIRAP*(CC)/*MyD88*(AG) vs *TIRAP*(CC)/*MyD88*(AA)	1.30	5.4 × 10^–2^
*TIRAP*(CT)/*MyD88*(AG) vs *TIRAP*(CC)/*MyD88*(AA)	0.20	9.8 × 10^–9^
*IL1RL1*(AA)/*TIRAP*(CT) vs *IL1RL1*(CC)/*TIRAP*(CC)	0.10	2.8 × 10^–17^
*IL1RL1*(AA)/*TIRAP*(CC) vs *IL1RL1*(CC)/*TIRAP*(CC)	0.61	2.2 × 10^–2^
*IL1RL1*(AA)/*MyD88*(AA)/*TIRAP*(CC) vs *IL1RL1*(CC)/*MyD88*(AA)/*TIRAP*(CC)	0.48	1.4 × 10^–2^
*IL1RL1*(AA)/*MyD88*(AA)/*TIRAP*(CT) vs *IL1RL1*(CC)/*MyD88*(AA)/*TIRAP*(CC)	0.14	1.4 × 10^–8^

*OR* odds ratio estimated by Fisher’s exact test; vs, withinlocus comparisons; /, between loci interactions.

Finally, reduced expression levels of four inflammatory mediators (IL-6, COX2, TNF-α, and IL-1β) were detected in patients with the *H. pylori*-resistant genotypes *IL1RL1*(AA)/*TIRAP*(CC); *IL1RL1*(AA)/*MyD88*(AA); and *IL1RL1*(AA)/*MyD88*(AA)/*TIRAP*(CT) vs I*L1LR1*(CC)/*MyD88*(AA)/*TIRAP*(CC) (Fig. 1b–d).

To the best of our knowledge, the present study is the first to describe the role of *MyD88*, *TIRAP*, and *IL1RL1* in the context of host resistance to *H. pylori* infection. A previous study by the same authors describes the interaction between *MyD88* and *TIRAP* and concludes that *MyD88* alone does not confer resistance to *H. pylori*, while the two genes do interact when in the double heterozygous combination (AG/CT; OR: 0.14.; *P*: 5.9 × 10^–13^)^18^. This result—confirmed in the present study (OR: 0.2.; *P*: 9.8 × 10^–9^)—is the unique detail linking the two studies.

### Nuclear magnetic resonance (NMR) analysis: cases versus controls

Binding of Il-33 to its receptor IL1RL1 may alter glucose and lipid metabolism^21,22^. Patients with type 2 diabetes or hypertriglyceridemia were therefore excluded. Thus, metabolome analysis was limited to blood samples from 59 cases and 17 controls. Representative proton spectra are shown in Supplementary Fig. S1.

**Figure 2:**
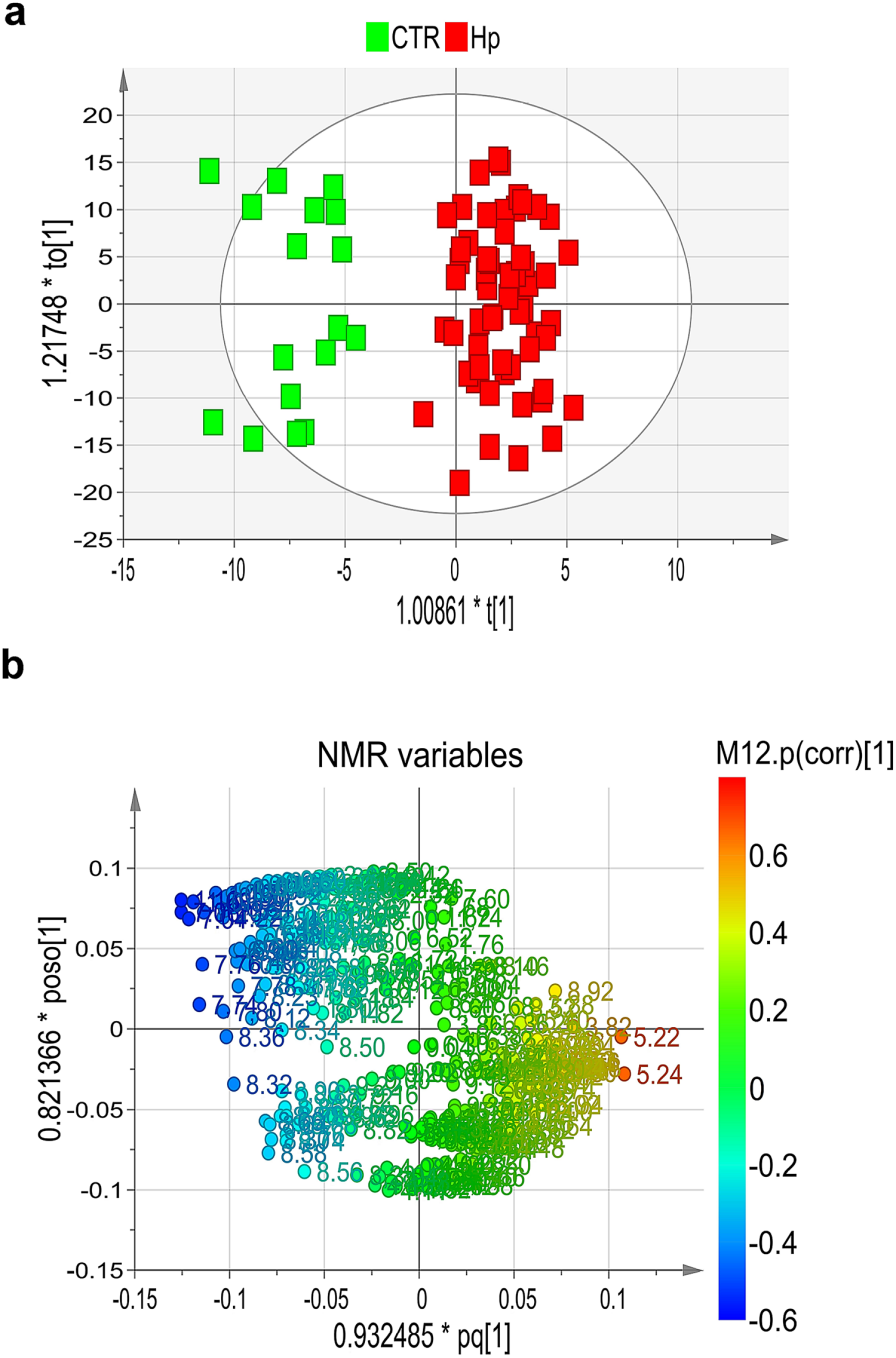
(**a**) Scores plot and (**b**) loadings plot of blood serum samples from cases and controls.

Resonances were assigned to metabolites by comparing 2D NMR data with literature and/or online databases. Unsupervised PCA models excluded the presence of outliers (data not shown). OPLS-DA (VIP value > 1; correlation loading values |*p*(corr)|> 0.5) and a regression model with one predictive and one orthogonal component (goodness of fit: R^2^ = 51%; power in prediction: Q^2^ = 37%; significance for CV-ANOVA: *P* = 0.000001) clearly differentiated cases (red squares) from controls (green squares) (Fig. 2a).

The scores plot of Fig. 2a shows a clear group separation along the predictive component between controls at negative values, and a dense cluster of cases placed mainly along t^1^ positive values. The second orthogonal component instead describes the variation within each group. The associated loadings plot describes the NMR variables responsible for group separation (Fig. 2b).

**Figure 3:**
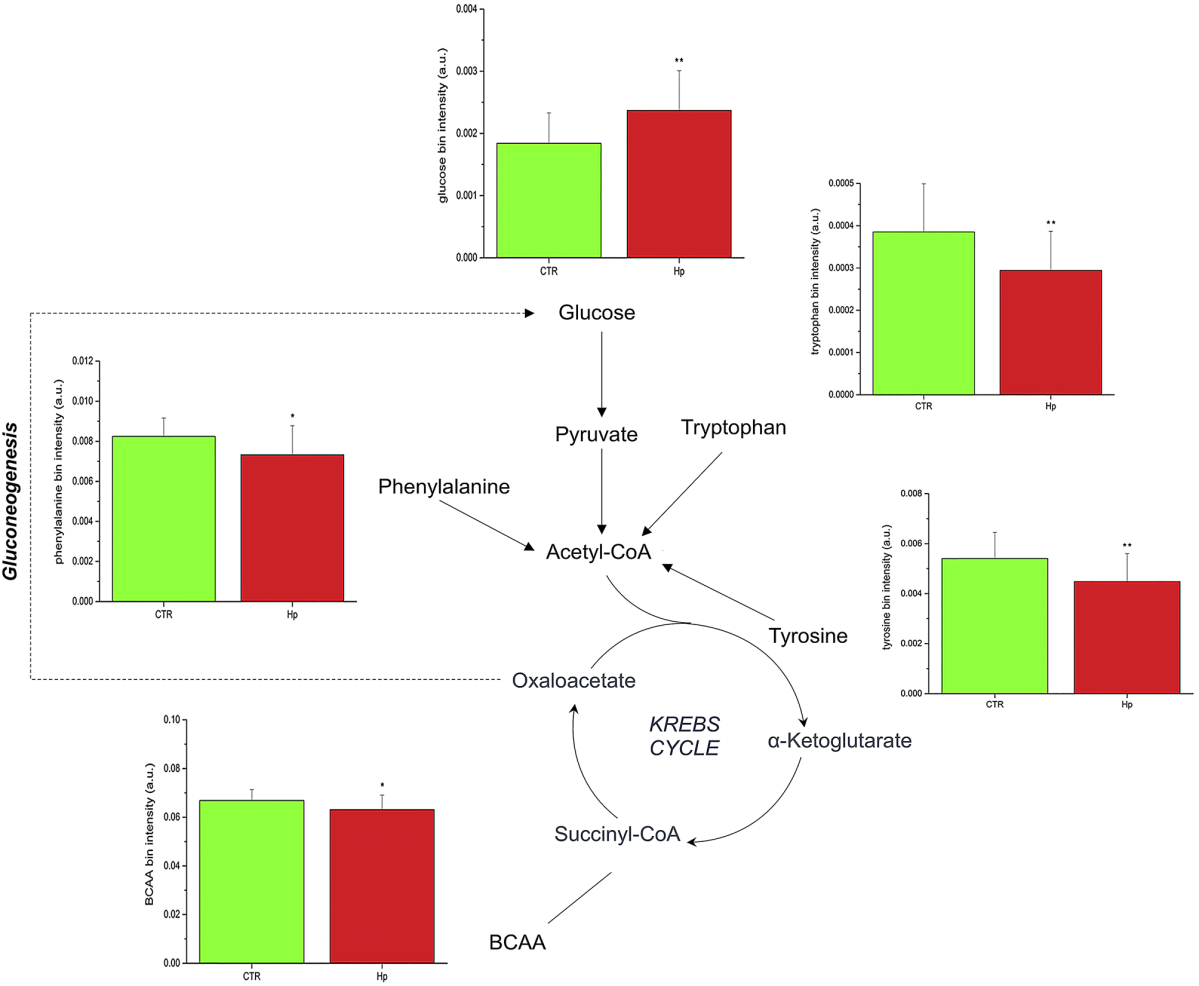
Expression levels and metabolic pathways of metabolites connected with Krebs cycle, glycolysis and gluconeogenesis.

**Figure 4:**
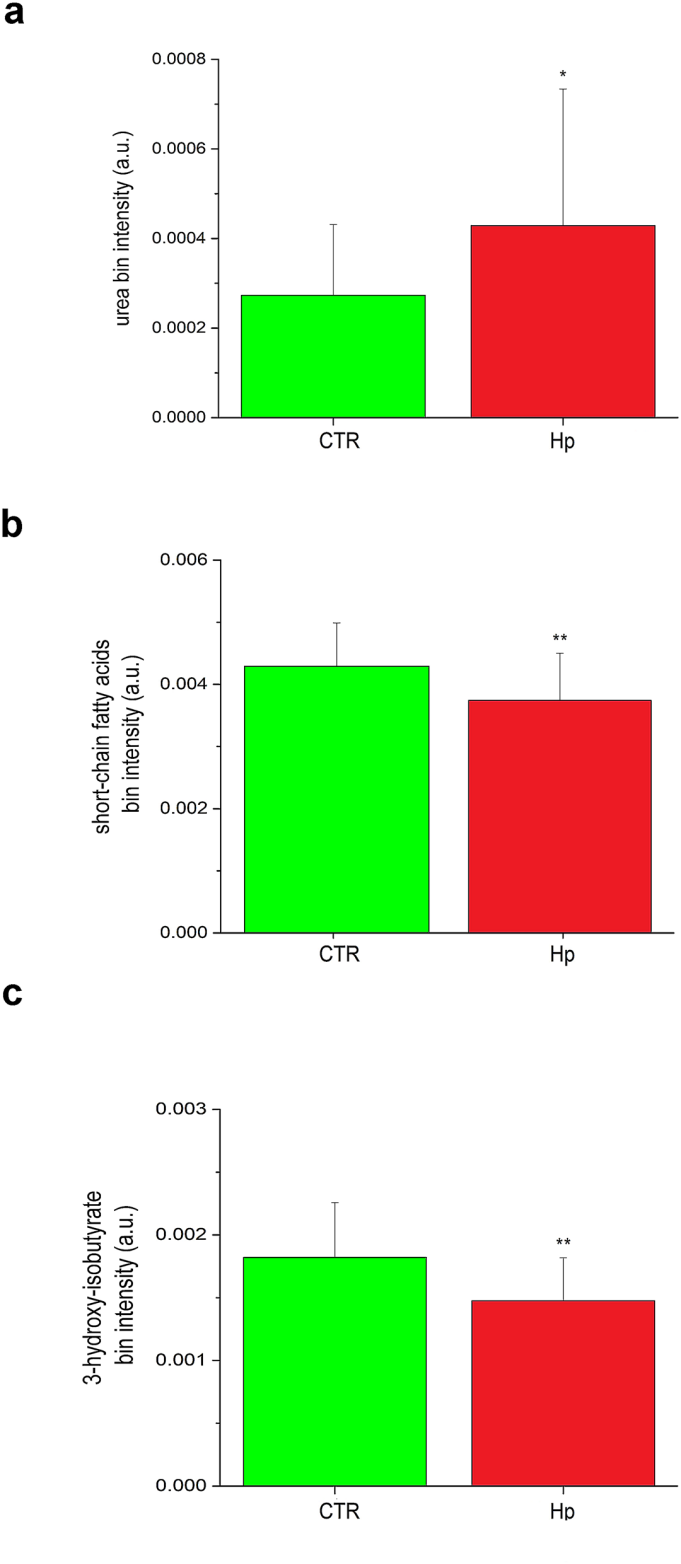
Expression levels of: (**a**) Urea, (**b**) Short chain fatty acids and (**c**) 3-Hydroxybutyrate detected in patients and controls.

The control group is characterized by high levels of tyrosine, tryptophan, phenylalanine, branched-chain amino acids (BCAA) (valine, leucine and isoleucine), 3-hydroxybutirate, short chain fatty acids (SCFAs) and methyl-histidine, while the case group displays high levels of glucose, glucose-1-phosphate, sucrose, urea, glycolipids, and niacinamide. In particular, except for glucose-1-phosphate, sucrose, and niacinamide, all the discriminating metabolites were statistically significant (Figs. 3 and 4).

**Figure 5:**
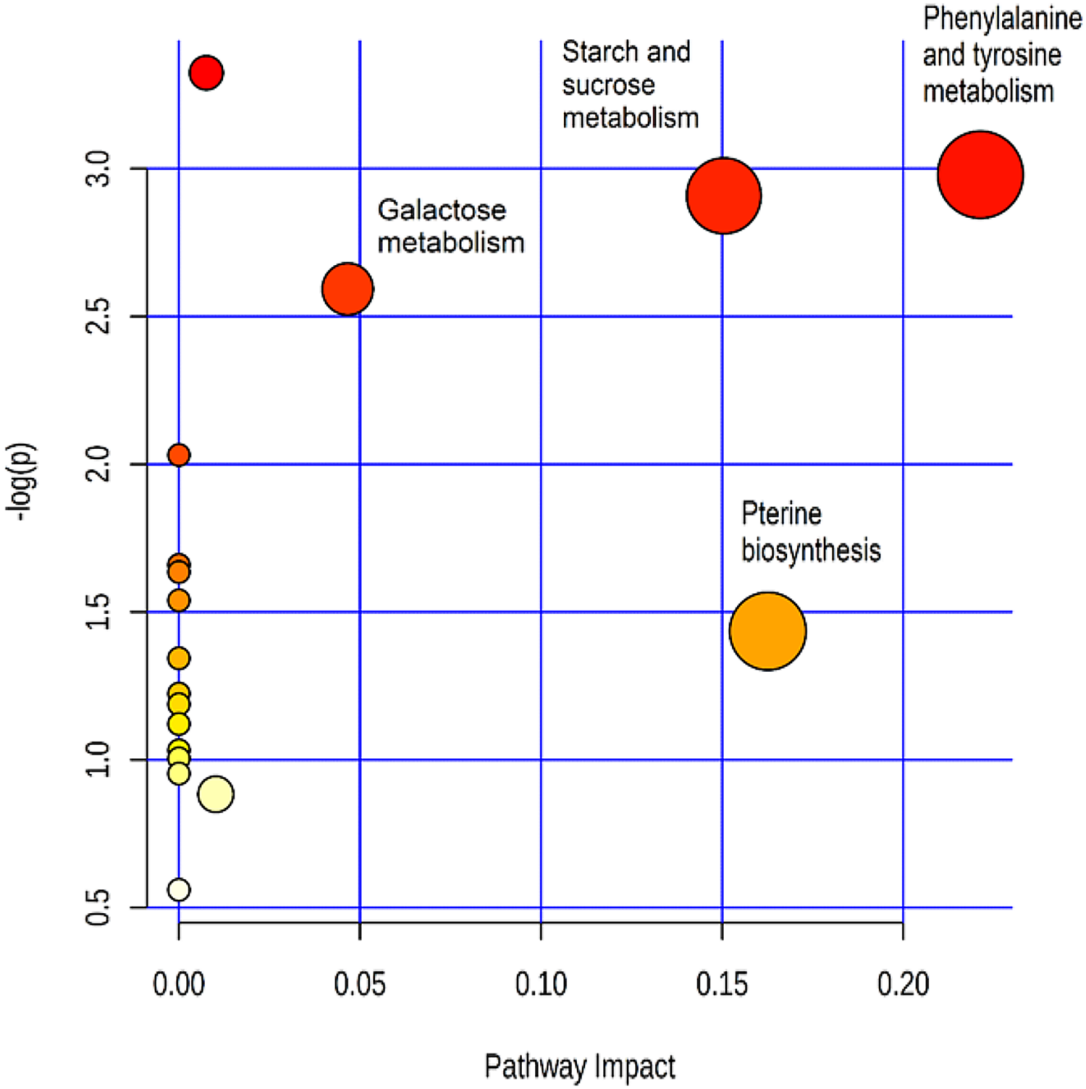
Impact and *P* value (− log(p)) of most representative metabolic pathways.

Further, pathway topology and biomarker analysis identified as significantly dysregulated the following metabolic pathways of phenylalanine, and tyrosine (*P* = 1.05 × 10^–3^; impact 0.22), pterine biosynthesis (*P* = 3.63 × 10^–2^; impact 0.16), starch and sucrose (*P* = 1.23 × 10^–3^; impact 0.15), and galactose metabolism (*P* = 2.57 × 10^–3^; impact 0.05) (Fig. 5).

Two differences are particularly relevant. First, the high level of urea displayed by the cases (Fig. 4) presumably reflects the increased need of urea by *H. pylori* for amino acid synthesis^23^ and neutralization of the nitrogen excess accumulated by deamination of amino acids^24^. Second, *H. pylori* infection and high glucose levels (Fig. 3)—acting synergistically^5^-cause oxidative stress, β-cell dysfunction, and altered insulin secretion^25^. Finally, it has been suggested that impaired folate metabolism caused by *H. pylori* infection may affect cognitive functions^26,27^. Thus, the presence of pterines (a substrate for folate production) detected among cases in this study supports the hypothesis that *H. pylori* may predispose to Alzheimer’s disease^3^.

### Nuclear magnetic resonance (NMR) analysis of cases

**Figure 6:**
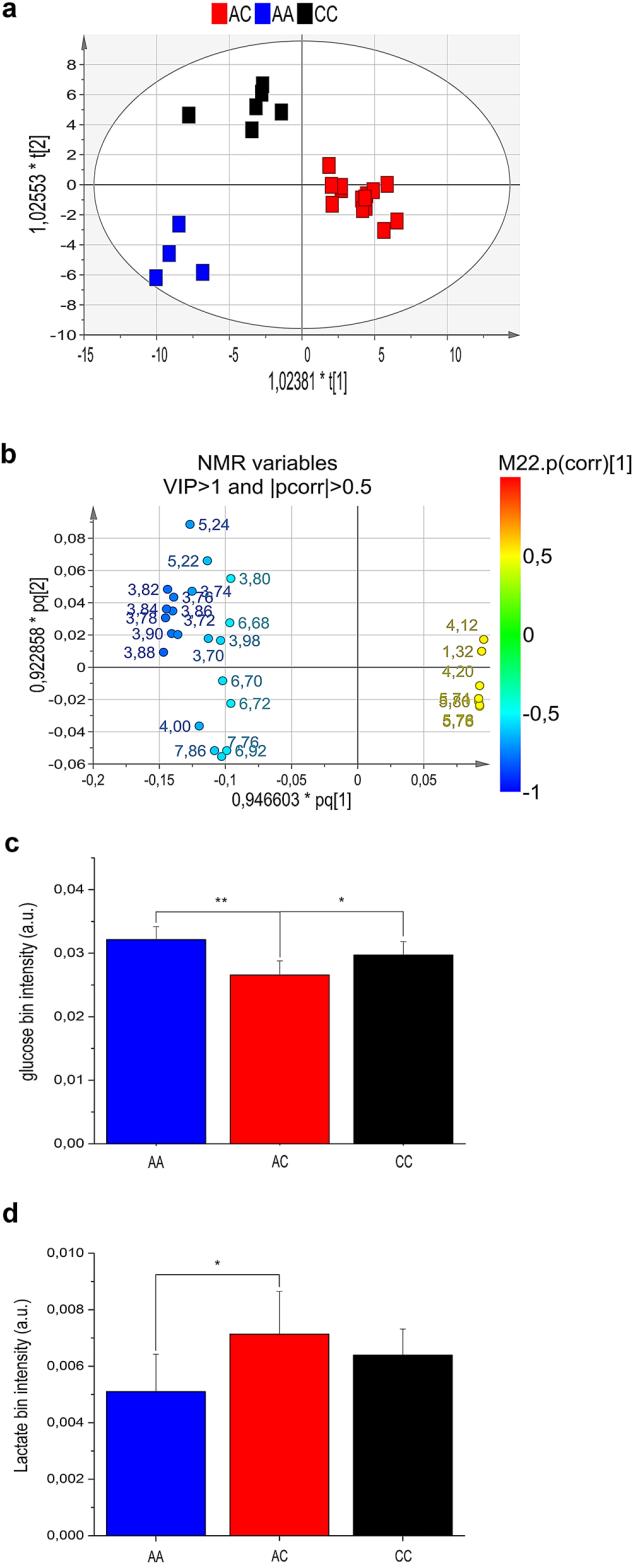
(**a**) Scores plot, (**b**) loadings plot, (**c**) glucose and (**d**) lactate levels of blood serum samples from patients homozygous at the MyD88 and TIRAP loci, but differing at the IL1RL1 locus.

Next aim was to identify potential metabolic differences between cases. This analysis was limited to the most representative class of cases (those homozygous at the *MYD88* and *TIRAP*, and heterozygous at the *IL1RL1* locus). For this purpose, it was built a regression model with two predictive components (R^2^ and Q^2^). The resulting scores plot differentiated heterozygous cases (*IL1RL1*A/C; n:14) from those homozygous (*IL1RL1*A/A (n:4) or *IL1RL1C/C *(n:6) (Fig. 6a)). The scores plot displays a main discrimination along the first predictive component between the heterozygous cases (red squares, located at t^1^ positive coordinates) and both the homozygous AA (blue squares) and CC (black squares) located at t^1^ negative coordinates. In addition, the second component t^2^ shows the separation between the two homozygous genotypes (AA and CC).

The loadings plot (Fig. 6b) shows the metabolites more expressed in the classes placed in the corresponding quarters of Fig. 6a, specifically the presence of high levels of lactate, urea and pyroglutamate in the AC group; high levels of glucose in that CC; and histidine in the AA group.

In particular, variables 4.12, 4.20, 1.32 (all originating from lactate) and 5.74–5.78 (from urea) are more intense in the corresponding AC group, which is placed at positive coordinates of the t^1^ axes (first component) in the scores plot. On the contrary, the same metabolites resulted less expressed in the AA and the CC groups, which are all placed at the opposite side of the t^1^ axis, namely negative values of the first component. The variables 5.24–5.22, and from 3.70 to 3.90 (Fig. 6b)—all corresponding to glucose resonances- are highly expressed in the CC group, placed at the corresponding superimposed quarter in the scores plot (Fig. 6a). Finally, variables 4.00, 6.92, 7.76, 7.86 from histidine resonances indicate the higher expression of this metabolite in the AA class. Signals with VIP value > 1 and correlation loading values |*p*(corr)|> 0.5 were selected as most relevant in the model discrimination. The corresponding bin quantification of the statistically significant metabolites glucose, and lactate are reported in Fig. 6c,d.

**Figure 7:**
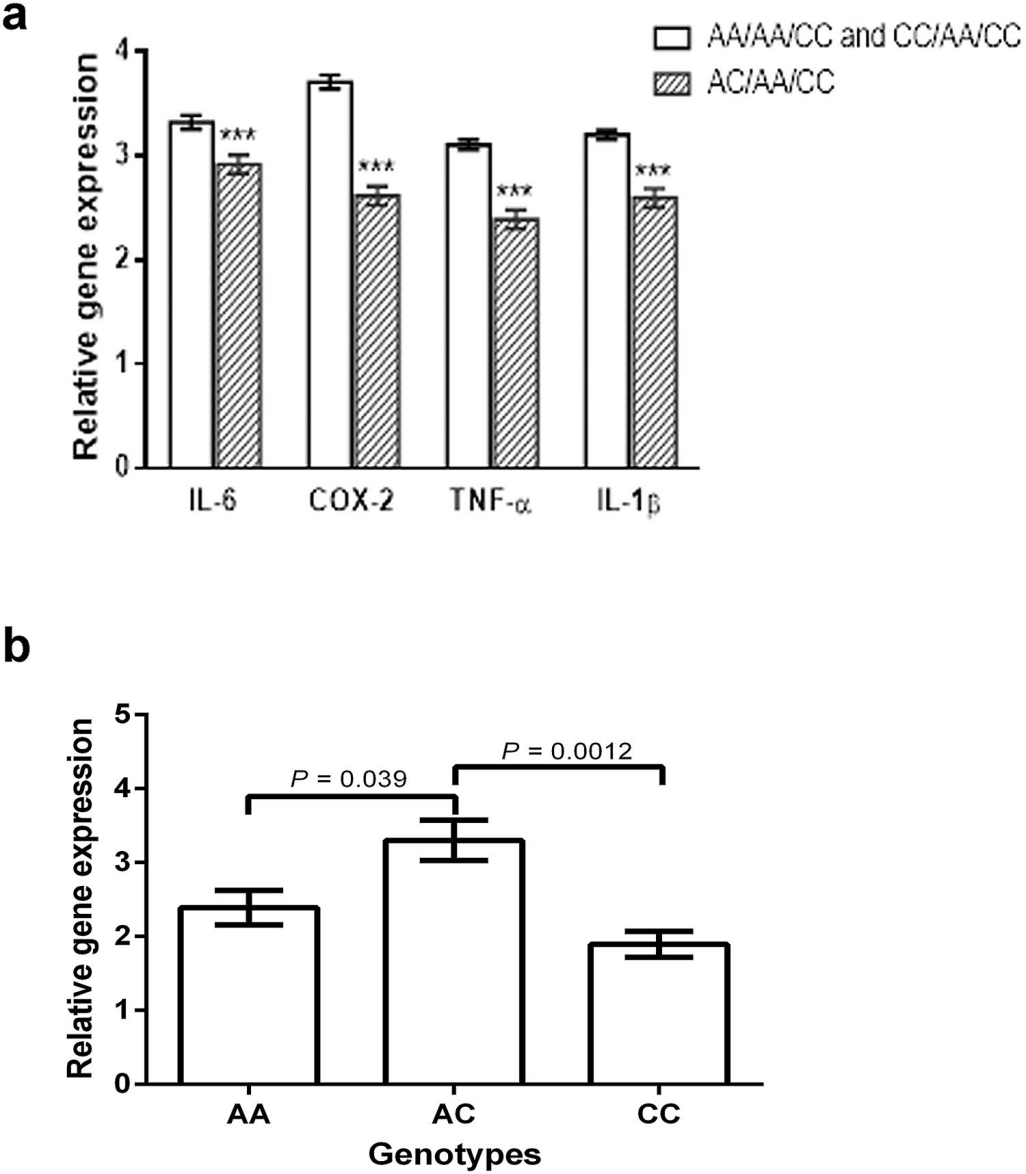
Expression levels of (**a**) IL-6, COX-2, TNF-α and IL-1β and (**b**) IL1RL1 in patients homozygous at the MyD88 and TIRAP loci, but differing at the IL1RL1 locus.

Finally, AC cases display low levels of IL-6, COX-2, TNF-α, IL-1β, and instead high level of IL1RL1, compared to homozygous cases (Fig. 7a,b).

## Discussion

There is growing evidence that genes rarely work alone^28,29^. More frequently, proteins tend to assemble into a complex, known as “cluster”, or “gene network”^30^. A gene cluster occurs more frequently between genes that physically interact or are members of the same biochemical pathway^28,31^. To detect the interaction between *MyD88* and *TIRAP* against *H. pylori* infection^18^, we built up on the notion that the MyD88 and TIRAP proteins co-immune precipitate^14^. Here, to detect a potential third partner of *MyD88* and *TIRAP*, we used as probe the notion that *IL1RL1*, *MyD88,* and *TIRAP* are members of the same biochemical pathway^13^. The propensity of these genes to interact was then confirmed by the STRING tool (Fig. 1a).

Bateson defined epistasis as the phenomenon of a gene altering the phenotype of another gene^32^. Later, Fisher used the same term to describe two or more genes interacting non-additively^33^. The gene interactions described in this study conform to the statistical definition of Fisher as well as to that functional of Bateson. When tested individually, *TIRAP* (OR: 0.50; *P*: 3.7 × 10^–6^) and *IL1RL1* (OR: 0.59; *P*: 1.2 × 10^–4^)—but not *MyD88* (OR: 0.98; P: 0.95) – confer resistance to *H. pylori* infection (Table 1). However, specific combinations of *MyD88 and TIRAP* confer protection (OR: 0.20; *P*: 9.8 × 10^–9^) (Table 2). Robust interactions have also been observed between specific combinations of *IL1RL1* and *TIRAP* (OR: 0.10; *P*: 2.8 × 10^–17^) and between *IL1RL1, MyD88* and *TIRAP* (OR: 0.14; *P*: 1.4 × 10^–8^) (Table 2).

The marked differences noticed between metabolic profiles of cases and controls demand comments and plausible interpretations. BCAAs are present at low levels in cases. Pathway analysis shows that these molecules can generate glucose via gluconeogenesis (Fig. 3). Their reduced levels in patients may thus be explained assuming that BCCAs are depleted to secure the increased request of glucose associated with the response to *H. pylori* infection (Fig. 3). The high impact of the phenylalanine/tyrosine (impact 0.22; *P* = 1.05 × 10^–3^;) and starch/sucrose (impact 0.15; *P* = 1.23 × 10^–3^) pathways concur with the proposed explanation (Fig. 5).

Major metabolic differences between cases and controls also involve inflammation. Patients infected with *H. pylori* show an excess of glucose (Fig. 3) and low levels of the ketone body 3-hydroxybutyrate and SCFAs (Fig. 4); the latter two molecules both inhibit NLRP3 activation^34^. This setting suggests that part of the excess of glucose might be converted to palmitate, which suppresses AMP-activated protein kinase, leading to ROS production and activation of the NLRP3 inflammasome^35,36^. In this context it seems plausible suggesting that the ketone body 3-hydroxybutyrate and SCFAs might be mobilized to counteract NLRP3 activation^37,38^. The proposed interpretation convincingly explains the reduced levels of SCFAs and 3-hydroxybutyrate.

Members of the TIR superfamily start the immune response by activating transcription of NF-kB and secretion of pro-inflammatory cytokines^39^. However, to prevent detrimental effects, inflammation needs to be tempered. This key function is assumed by *IL1RL1*. While almost all members of the TIR superfamily induce a TH1 (pro-inflammatory) response, *IL1RL1* (though member of the same family) inhibits the adaptors MyD88 and TIRAP and activates a TH2 (anti-inflammatory) response^39^, characterized by production of regulatory T cells (Treg), activation of the glucose transport gene *GLUT1*, that enhances glucose uptake and production of lactate^40–42^. In turn, lactate contributes to curb inflammation^43^ by reducing the levels of the pro-inflammatory cytokines IL-1β, TNF-α, and IL-6^44^, while *H. pylori* senses lactate through the chemoattractant receptor TlpC^45^.

The above data on the anti-inflammatory role of *IL1RL1* well support our suggestion that heterozygosity at the locus *IL1RL1* is associated with reduced inflammation in *H. pylori*-infected patients. This conclusion is based on several independent lines of evidence: a regression model with two predictive components clearly separate patients heterozygous at the *IL1RL1* locus (AC) from those homozygous (CC or AA) at the same locus (Fig. 6a; R^2^ = 43; Q^2^ = 5%).

The results of the OPLS-DA analysis (R^2^ = 43; Q^2^ = 5%) were confirmed by the independent procedure of probability calculus, which established that the probability that the patients in Fig. 6a cluster together by chance is 1.8 × 10^–12^ (see “Methods” section). This result shows the under-appreciated opportunity offered by metabolomics to reach solid conclusions enrolling a limited number of patients.

The AC patients are characterized by high levels of lactate (Fig. 6d), and IL1RL1 (Fig. 7b) (both associated with anti-inflammatory activity; see above)—and low levels of the pro-inflammatory molecules IL-1β, TNF-α, COX-2, and IL-6 (Fig. 7a). It is also cogent noting that the anti-inflammatory activity associated with the *IL1RL1*-AC genotype prescinds from the genotypes at the *MyD88* and *TIRAP* loci (Table 1).

*MyD88, TIRAP*, and *IL1RL1* well describe the elegant flexibility characterizing gene clusters. The majority of the TIR family members induce inflammation^34^. However, since an excess of inflammation is detrimental, the family includes *IL1RL1*, that curbs inflammation sequestering the pro-inflammatory adaptors *MyD88* and *TIRAP*^39^. Thus, to gain adaptability, gene clusters include members exerting opposite functions and network genetics engages Mendelian genetics. *IL1RL1*, independent from *MyD88* and *TIRAP* (Table 2), can finely control inflammation through the advantage of heterozygotes (the phenomenon describing the higher fitness of the heterozygous genotype compared to both homozygous genotypes), the dominant force maintaining genetic variation in the populations^46,47^ and common diseases variants^48^.

A lateral result from this study, is that several metabolic pathways dysregulated by *H. pylori*—tyrosine, starch/sucrose and pterines metabolisms (Fig. 5)—have recently been reported to be dysregulated also in patients with Alzheimer’s disease^49–52^. These findings support the hypothesis that *H. pylori* may predispose to Alzheimer’s disease^3^.

In summary, compared to single locus association studies, analysis of gene clusters extends results to several loci, increases the statistical power, and uncovers novel information about metabolic pathways associated with diseases^53^. Further, our data show that the combined analysis of genes and metabolites leads to results (such as patients subtyping on the basis of their inflammation levels), that the gene approach alone does not reach.

This study, which highlights a crosstalk between genes—in particular SNPs—and metabolites, could represent the basis for developing personal and specific therapeutic treatments. By this way, the proposed approach could be considered as “new alternative” to the well-known antimicrobial peptides, in the case of resistant strains such as *Staphylococcus epidermidis*^54^ or, to the specific immunomodulatory methods for coeliac disease^55^.

Whether *IL1RL1* and lactate might represent clinically useful biomarkers of the inflammation remains to be investigated.

## Methods

### Cases and controls

Cases and controls are the same used in the previous study (at least those still available)^18^. Patients with dysmetabolic diseases (type 2 diabetes or obesity) were excluded. Cases (498) were positive by the bacteriological, hematoxylin–eosin, and PCR tests for *H. pylori*. Controls (702) were participants negative to the above tests and, to exclude past infection, to the *H. pylori*-specific IgG antibody test (Abcam, Cambridge, UK; code ab108736)^18^.

The study has been approved by the Ethics Committee of Villa Betania Hospital, and carried out in accordance with relevant guidelines and regulations (Declaration of Helsinki). In addition, the informed consent has been obtained from all participants.

### Genotyping

Probes and TaqMan genotyping master mix were from Applied Biosystems (Life Technologies, Monza, Italy). Probes were specific for the following polymorphic sites (SNPs): IL1RL1 rs11123923, MyD88 rs6853 and TIRAP rs8177374. The PCR program was as described^18^. To confirm genotyping accuracy, PCR products representing 10% of the sample population were sequenced. ORs and 95% confidence intervals were calculated by Fisher’s exact test using the statistical package GraphPad Prism version 5 (GraphPad, La Jolla, CA, USA).

### Quantitative real-time PCR

RNA samples were reverse transcribed with the High-Capacity cDNA Reverse Transcription Kit (Applied Biosystem, Thermo Fisher Scientific Inc, Milan, Italy). Real-time PCR of *IL-6, COX-2, TNF-α* and *IL-1β* was carried out as described^18^. The expression level of *IL1RL1* was measured using the TaqMan Gene Expression Assay (Hs00249384_m1; Life Technologies, Monza, Italy), and TaqMan PCR master 2X reagent (Applied Biosystem, Thermo Fisher Scientific Inc,, Milan, Italy). The Applied Biosystem iCycler was used according to the manufacturer’s instructions. PCR reactions were carried out in triplicate; expression values were calculated according to 2^−∆∆Ct^ method and normalized against human glyceraldehydes-3-phophate dehydrogenase (GAPDH) levels. As “calibrator”, we used a negative (control) sample.

The statistical analysis was carried out according to the two-way ANOVA using the statistical package GraphPad Prism version 5 (GraphPad, La Jolla, CA, USA).

### Protein network analysis

*IL1RL1* was identified as third partner of *MyD88* and *TIRAP* using the STRING database (https://string-db.org). The level of confidence and the maximum number of interacting proteins were set at 0.4 and 5, respectively.

### Metabolites extraction

Metabolites were extracted from blood samples (59 cases and 17 controls) as described^56^. Briefly, 2.5 mL of chloroform: methanol: dd H_2_O (1:1:1.3) mixture were added to 500 µL of individual blood samples and rapidly centrifuged (410 rpm; 20 min at 4 °C). The upper polar phase was collected and vacuum -dried at 30 °C using the rotational vacuum concentrator (model RVC 2-18 CD plus; Martin Christ Gefriertrocknungsanlagen GmbH, Osterode am Harz, Germany).

Dried samples were suspended in 630 µL of phosphate buffer saline (PBS) plus 70 µL of deuterated solvent (containing 0.1 mM sodium 3-trimethylsilyl [2,2,3,3-^2^H_4_] propionate (TSP) as a chemical shift reference for ^1^H spectra). Deuterated solvent was added to obtain a field-frequency lock. The final individual sample volume was 700 µL. All reagents were from Sigma-Aldrich S.r.l. Milan, Italy.

### NMR spectroscopy

NMR spectra were recorded on a Bruker Avance III–600 MHz spectrometer (Bruker BioSpin GmbH, Rheinstetten, Germany) equipped with a TCI CryoProbe™, fitted with a gradient along the Z-axis, at a probe temperature of 300 K (27 °C).

Profile analysis and metabolites identification were determined from one- (1D), and two-dimensional (2D) spectra.

For further details, see Supplementary Methods.

### Multivariate data analysis

The 0.60–9.40 ppm spectral area of blood aqueous extracts underwent bucketing, and each region of 0.02-ppm width was integrated by using the AMIX 3.9.15 software (Bruker Biospin GmbH, Rheinstetten, Germany). For further details, see Supplementary Methods.

### Principal component analysis (PCA) and orthogonal projection to latent structures discriminant analysis (OPLS–DA)

PCA^57^ and OPLS-DA^58^ were carried out with the SIMCA P+14 package (Umetrics, Umeå, Sweden). Data trends and the presence of possible outliers were evaluated by PCA, while OPLS-DA was used to better define clustering and metabolic variation. For further details, see Supplementary Methods.

The results of the OPLS-DA analysis were confirmed by the independent procedure of the probability calculus. The probability that the 4 patients AA cluster together by chance is (0.33)^4^ = 10^–2^; that the 6 patients CC cluster together by chance is (0.33)^6^ = 10^–3^; the probability that the 14 patients AC cluster together by chance is (0.33)^14^ = 1.8 × 10^–7^. The probability that the three events occur concurrently by chance is 10^–2^ × 10^–3^ × 1.8 × 10^–7^ = 1.8 × 10^–12^.

### Pathway analysis

Pathway topology and biomarker analysis of discriminating metabolites were carried out by using MetaboAnalyst 4.0.^59^. For further details, see Supplementary Methods.

## Supplementary information

Supplementary Information.

## Data Availability

The authors declare that all the data supporting the findings of this study are included in this paper and its Supplementary Information files, and also are available from the corresponding author upon reasonable request.
