# Supplementary material for: Interaction between MyD88, TIRAP and IL1RL1 against Helicobacter pylori infection
Source: Sci Rep. 2020 Sep 28;10:15831. doi: 10.1038/s41598-020-72974-9 (PMC7522988; doi:10.1038/s41598-020-72974-9)
Supplement: Supplementary file 1 — Supplementary Information. [file 41598_2020_72974_MOESM1_ESM.pdf]

Supplementary Information

Title: Interaction between *MyD88, TIRAP* and *IL1RL1* against *Helicobacter pylori* infection.

Andrea Fulgione[1,2,+], Marina Papaianni[1,+], Paola Cuomo[1], Debora Paris[3], Marco Romano[4], Concetta Tuccillo[4], Letizia Palomba[5], Chiara Medaglia[6], Massimiliano De Seta[7], Nicolino Esposito[7], Andrea Motta[3], Antonio Iannelli[8,9,10], Domenico Iannelli[1,*], Rosanna Capparelli[1,+]

[1]Department of Agriculture Sciences, University of Naples “Federico II”, via Università, 100 - 80055 Portici, Naples, Italy.

[2]Istituto Zooprofilattico Sperimentale del Mezzogiorno, via Salute, 2 – 80055 Portici, Naples, Italy.

[3]Institute of Biomolecular Chemistry, National Research Council, via Campi Flegrei, 34 - 80078 Pozzuoli, Naples, Italy.

[4]Hepatogastroenterology Unit, Department of Precision Medicine, University of Campania "Luigi Vanvitelli", via Pansini, 5- 80131 Naples, Italy.

[5]Department of Biomolecular Sciences, University of Urbino “Carlo Bo”, via Santa Chiara, 27 - 61029 Urbino, Italy

[6]Department of Microbiology and Molecular Medicine, University of Geneva Medical School, rue du Général-Dufour, 24 - 1211 Genève 4, Switzerland.

[7]Fondazione Evangelica Betania, via Argine, 604 - 80147 Naples, Italy.

[8]Université Côte d’Azur, Campus Valrose, Batiment L, Avenue de Valrose, 28 - 06108 Nice CEDEX 2, France;

[9]Centre Hospitalier Universitaire de Nice - Digestive Surgery and Liver Transplantation Unit, Archet 2 Hospital, route Saint-Antoine de Ginestière 151, CS 23079 - 06202 Nice CEDEX 3, France;

[10]Inserm, U1065, Team 8 “Hepatic complications of obesity and alcohol”, route Saint Antoine de Ginestière 151, BP 2 3194 - 06204 Nice CEDEX 3, France.

[+] These authors contributed equally to this work

*** Corresponding Author**

Domenico Iannelli. Department of Agriculture Sciences, University of Naples “Federico II”, Portici, Naples, Italy. Tel.: +39 081 2539276. E-mail: domenico.iannelli1935@gmail.com

Supplementary Table S1. DbSNP ID of IL1RL1 identified by sequencing.

rs985523; rs873022; rs78841971; rs78197197; rs7596970; rs72823646; rs6752482; rs6751977; rs6751967; rs6749114; rs6734742; rs6719130; rs6704565; rs6543119; rs62152663; rs62152662; rs62152661; rs59247511; rs55927292; rs4988958; rs4988957; rs4988956; rs4988955; rs3821204; rs3771179; rs3771177; rs3771175; rs3732129; rs34210856; rs3214363; rs2160203; rs201145282; rs200544577; rs1946131; rs1921622; rs1861246; rs1861245; rs182823905; rs17696376; rs17696274; rs17027006; rs148040699; rs1420101; rs141010284; rs140594705; rs139756621; rs138892317; rs13431828; rs13424006; rs13408661; rs13408569; rs13029918; rs13028993; rs13022799; rs13017455; rs13016771; rs13014044; rs13007819; rs13007174; rs12999542; rs12999517; rs12996097; rs12989197; rs12905; rs12712142; rs12469506; rs116953957; rs114989917; rs11123923; rs1054096; rs1041973; rs10208293; rs10206753; rs10204137; rs10197862; rs10192157; rs10192036; rs10185897; rs10173081; rs10173081; rs10185897; rs10192036; rs10192157; rs10197862; rs10204137; rs10206753; rs10208293; rs1041973; rs1054096; rs11123923; rs114989917; rs116953957; rs12469506; rs12712142; rs12905; rs12989197; rs12996097; rs12999517; rs12999542; rs13001714; rs13007174; rs13007819; rs13014044; rs13016771; rs13017455; rs13022799; rs13028993; rs13029918; rs13408569; rs13408661; rs13424006; rs13431828; rs138892317; rs139756621; rs140594705; rs141010284; rs1420101; rs148040699; rs17027006; rs17696274; rs17696376; rs182823905; rs1861245; rs1861246; rs1921622; rs1946131; rs200544577; rs201145282; rs2160203; rs3214363; rs34210856; rs3732129; rs3771175; rs3771177; rs3771179; rs3821204; rs4988955; rs4988956; rs4988957; rs4988958; rs55927292; rs59247511; rs62152661; rs62152662; rs62152663; rs6543119; rs6704565; rs6719130; rs6734742; rs6749114; rs6751967; rs6751977; rs6752482; rs72823646; rs7596970; rs78197197; rs78841971; rs873022; rs985523.

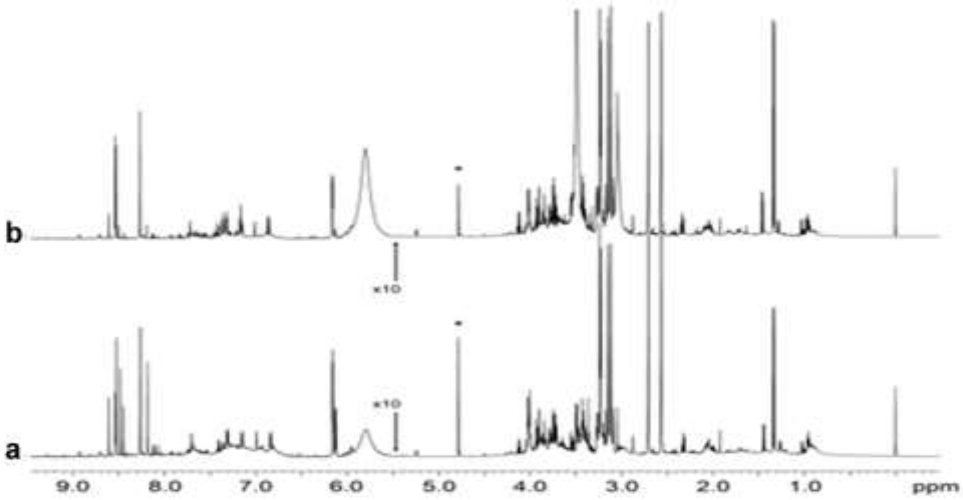

Supplementary Figure S1. NMR spectra of blood samples. Representative 1D 1H spectra of (a) an healthy subject, and (b) one *H. pylori*-infected. The region between 9.0 and 5.5 ppm has a 10-fold vertical expansion. All signals were assigned to single metabolites by resorting to 2D NMR experiments or referring to published data. Absorption is plotted on the y-axis, and magnetic field strength on the x-axis. The asterisk marks the residual water signal.

Supplementary Methods. NMR spectroscopy, Multivariate data analysis, Principal Component Analysis (PCA) and Orthogonal Projection to Latent Structures Discriminant Analysis (OPLS–DA) and pathway analysis.

NMR spectroscopy.

NMR spectra were recorded on a Bruker Avance III–600 MHz spectrometer (Bruker BioSpin GmbH, Rheinstetten, Germany) equipped with a TCI CryoProbeTM, fitted with a gradient along the Z–axis, at a probe temperature of 300 K (27°C). In 1D experiments, the excitation sculpting sequence was used to suppress the water resonance[1]. Prior to Fourier transformation, an exponential multiplication of 0.8 Hz was applied. Two-dimensional (2D) clean total correlation spectroscopy (TOCSY)[2] was carried out by selected a standard pulse sequence with a spin-lock period of 64 ms, achieved with the MLEV–17 pulse sequence, and incorporating the excitation sculpting sequence for water suppression. Prior to Fourier transformation, a Lorentz-to-Gauss window with different parameters was applied for both t1 and t2 dimensions for all the experiments. Spectra were referred to internal 0.1 mM TSP, assumed to resonate at $\delta = 0.00$ ppm. Natural abundance 1H–13C heteronuclear single quantum coherence (HSQC) spectra were detected acquired using an echo-antiecho phase sensitive pulse sequence with adiabatic pulses and pre-saturation for decoupling and water suppression, respectively. Before Fourier transformation, a shifted cosine window was applied in both dimensions. Linear prediction was also applied to extend the data to twice its length in t1. HSQC spectra were referred to the α-glucose doublet resonating at 5.24 ppm for 1H, and 93.10 ppm for 13C.

Multivariate data analysis.

The 0.60–9.40 ppm spectral area of blood aqueous extracts was automatically data reduced to integrated regions (buckets) of 0.02-ppm width using the AMIX 3.9.15 software (Bruker Biospin GmbH, Rheinstetten, Germany). The residual water resonance (4.50–5.06 ppm) was not included, and each bucket was normalized to the total spectrum area. The obtained data matrix was analysed with multivariate statistical regressions to discriminate and classify the NMR profiles.

Principal Component Analysis (PCA) and Orthogonal Projection to Latent Structures Discriminant Analysis (OPLS–DA).

Principal Component Analysis (PCA)[3] and Orthogonal Projection to Latent Structures Discriminant Analysis (OPLS–DA)[4] were carried out with the SIMCA P+14 package (Umetrics, Umeå, Sweden). Unit variance scaling was selected as data pre-treatment for both analyses. PCA was carried out to evaluate data trends (data not shown), while OPLS-DA better defined clustering and correlated the metabolic variations to pathophysiological changes. The 7-fold cross–validation and permutation tests (800 repeats) were carried out to evaluate the possible overfit of the OPLS-DA proposed models. The significance of OPLS-DA was assessed by the regression correlation coefficient R2 and the cross-validate correlation coefficient Q2. Shapiro-Wilk and D'Agostino K squared test and non-parametric Kruskal-Wallis Anova test were performed using OriginPro 9.1 software package (OriginLab Corporation, Northampton, USA).

Signal variations were presented as bin intensities normalized to the total area of each spectrum. Results were considered statistically significant at $p < 0.05$.

Pathway Analysis.

Pathway topology and biomarker analysis on selected and more representative discriminating metabolites were carried out by using MetaboAnalyst 4.0.[5]. The importance of each pathway was evaluated through the Pathway Impact. We selected the Homo sapiens (Small Molecule Pathway Database, http://smpdb.ca/) library, and Global Test and Relative Betweenness Centrality were chosen for pathway enrichment analysis and pathway topological analysis, respectively. Metabolites were identified setting VIP values > 1 in class discrimination and correlation values |pq[corr]| > 0.5 as parameters.

References

1. Hwang, T. L. & Shaka, A. J. Water Suppression That Works. Excitation Sculpting Using Arbitrary Wave-Forms and Pulsed-Field Gradients. *J. Magn. Reson. Ser. A* **112**, 275–279 (1995).
2. Griesinger, C., Otting, G., Wuethrich, K. & Ernst, R. R. Clean TOCSY for proton spin system identification in macromolecules. *J. Am. Chem. Soc.* **110**, 7870–7872 (1988).
3. Eriksson, L., Byrne, T., Johansson, E., Trygg, J. & Vikström, C. *Multi- and megavariate data analysis : basic principles and applications*.
4. Trygg, J. & Wold, S. Orthogonal projections to latent structures (O-PLS). *J. Chemom.* **16**, 119–128 (2002).
5. Chong, J. *et al.* MetaboAnalyst 4.0: towards more transparent and integrative metabolomics analysis. *Nucleic Acids Res.* **46**, W486–W494 (2018).
